# Association between age at menarche and bone mineral density in postmenopausal women

**DOI:** 10.1186/s13018-023-03520-2

**Published:** 2023-01-17

**Authors:** Yimei Yang, Shanshan Wang, Hui Cong

**Affiliations:** 1 Department of Obstetrics and Gynecology, Affiliated Hospital of Nantong University, Nantong, 226001 China; 2 Department of Blood Transfusion, Affiliated Hospital of Nantong University, #20 Xisi Road, Nantong, 226001 China; 3 Department of Laboratory Medicine, Affiliated Hospital of Nantong University, Nantong, 226001 China

**Keywords:** Menarche, Bone mineral density, Postmenopausal women, Osteoporosis, NHANES

## Abstract

**Background:**

Age at menarche (AAM) directly affects female estrogen levels, which play a vital role in bone metabolism. The exact relationship between bone mineral density (BMD) and AAM remains controversial. Thus, this study aimed to determine the association between AAM and lumbar spine (LS) BMD in postmenopausal women.

**Methods:**

Our data were based on the National Health and Nutrition Examination Survey (NHANES) 2011–2018. AAM was divided into three categories including ≤ 12, 13–15, and ≥ 16 years, and the ≤ 12 years old category was used as the reference group. To examine the association between AAM and LS BMD, we used three weighted linear regression models, Model 1 (without adjustment), Model 2 (with adjustment for age, race, and body mass index [BMI]), and Model 3 (with adjustment for all covariates).

**Results:**

This study included 1195 postmenopausal women aged 40–59 years. In the unadjusted model, a menarche age of ≥ 16 years compared with a menarche age of ≤ 12 years was associated with lower LS BMD (*β* = − 0.083, 95% CI − 0.117, − 0.048, *P* < 0.001). After adjusting for potential confounding factors, there was still a negative correlation in model 2 (*β* = − 0.078, 95% CI − 0.113, − 0.042, *P* < 0.001) and model 3 (*β* = − 0.065, 95% CI − 0.096, − 0.033, *P* < 0.001). Moreover, this significant relationship persisted after excluding participants who used female hormones (*β* = − 0.053, 95% CI − 0.089, − 0.016, *P* = 0.006).

**Conclusion:**

Our study found that postmenopausal women with a menarche age of ≥ 16 years had significantly lower LS BMD than that had by those with a menarche age of ≤ 12 years. As a result of this study, postmenopausal women with a late menarche age may have a higher risk of lumbar osteoporotic fractures and need better bone health care.

## Background

Osteoporosis is one of the most common diseases worldwide, and approximately nine million people sustain fractures every year due to osteoporosis [1–3]. According to predictions, osteoporotic fractures in the USA will triple due to aging [4–6]. Evidence has suggested that low bone mineral density (BMD) can increase the risk of fracture in both men and women [7–9]. Lumbar spine (LS) BMD is often measured using dual-energy X-ray absorptiometry (DXA) and has been incorporated into some clinical guidelines [10].

After menopause, the rate of bone mass loss increases, if women have risk factors for osteoporosis, then the process of bone destruction will exceed the process of bone formation. Therefore, the identification of risk factors for osteoporosis is crucial. Menarche is the beginning of a woman’s menstrual cycle and an important symbol of a woman’s reproductive stage. Age at menarche (AAM) directly affects the female estrogen levels, which play a vital role in bone metabolism. Despite extensive research in this field [11–17], the exact relationship between BMD and AAM remains controversial. In postmenopausal women, some studies suggested that early menarche was positively associated with high LS BMD, and late menarche was associated with decreased BMD and increased risk of osteoporosis [11–13], while others suggested no correlation between age at menarche and LS BMD [14–17]. Therefore, the aim of our study was to determine whether there was a correlation between AAM and LS BMD among postmenopausal women.

## Method

### Study population

The data were obtained from the National Health and Nutrition Examination Survey (NHANES), a survey designed to determine the health and nutritional status of American adults and children. An important feature of this survey is the combination of interviews and physical examinations for data collection. Demographic, socioeconomic, dietary, and health-related questions were asked during the NHANES interview. The examination part included medical, dental, physiological measurements, and laboratory tests [18]. Informed consent was obtained from each participant, and the Institutional Review Board of the National Center for Health Statistics approved the survey protocol.

**Fig. 1:**
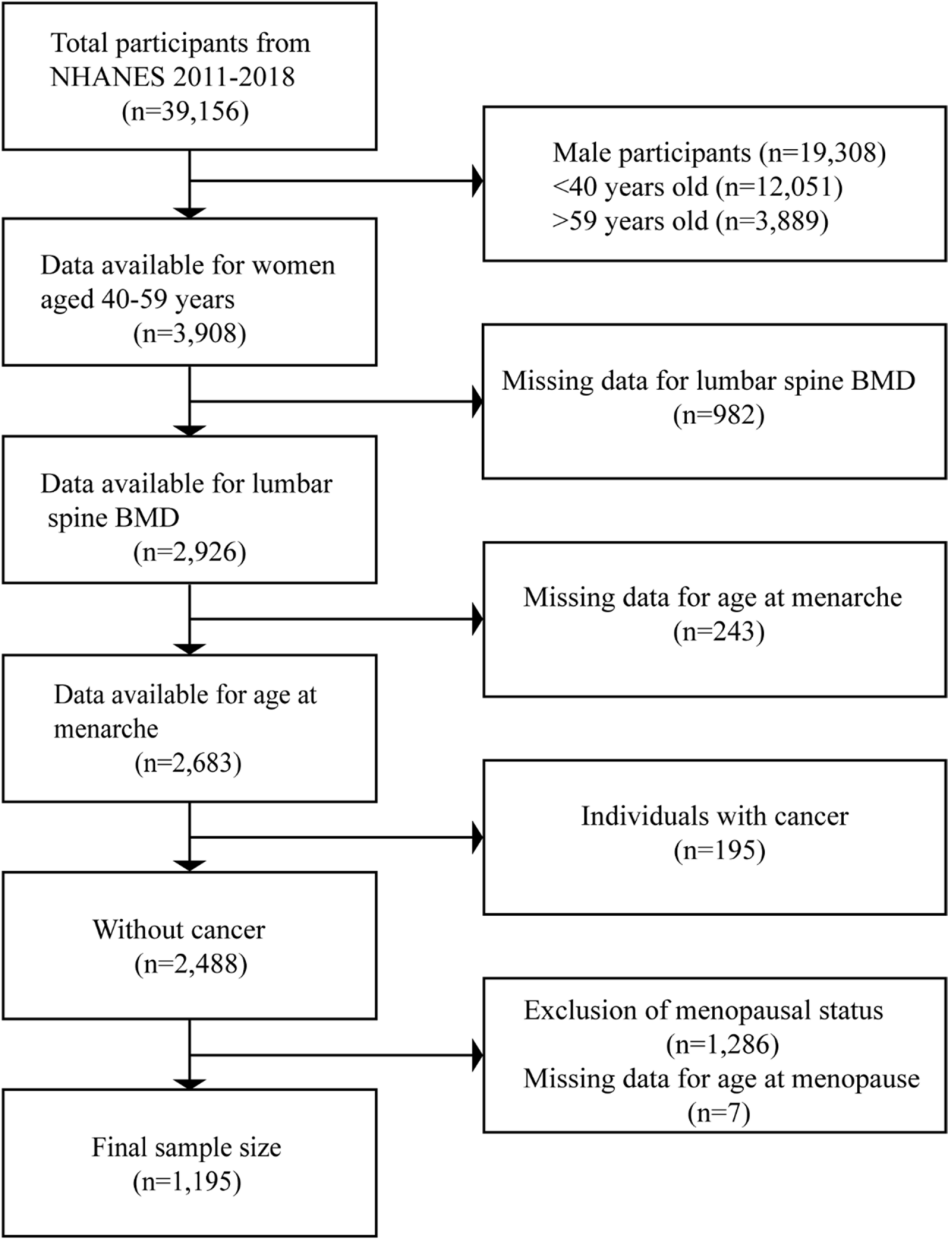
Flowchart of research participant selection

This study merged data from four continuous cycles (2011–2012, 2013–2014, 2015–2016, and 2017–2018). The NHANES project recruited 39,156 participants between 2011 and 2018. After excluding the data of individuals with missing main variables data and of individuals with cancer, a total of 1195 postmenopausal women aged 40–59 years remained and were included in this study (Fig. 1).

### Study variables

The study exposure is AAM. AAM was assessed using a reproductive health questionnaire in the NHANES 2011–2018 using the question “How old were you when you had your first menstrual period?” AAM was divided into three categories including ≤ 12, 13–15, and ≥ 16 years, and the ≤ 12 years old category was used as the reference group.

The outcome of this study is LS BMD. The measurement of LS BMD has been used for the evaluation and treatment of osteoporosis [19]. LS BMD was measured using DXA, which was performed by trained and certified radiology technologists [20].

Self-reported information about age, race, education level, family poverty income ratio (PIR), smoking behavior, alcohol consumption, reproductive health, parity, female hormone use, vigorous activities, moderate activities, arthritis, thyroid disease, liver disease, kidney disease, and diabetes were obtained. Family PIR was stratified into three levels: < 1.3 (low income), 1.3–3.5 (middle income), and ≥ 3.5 (high income) [21]. There were three categories of alcohol consumption including non-drinkers, moderate drinkers, and heavy drinkers. Women who drink moderately consumed less than two drinks each day, and women who drink heavily consumed two or more drinks per day [22]. Furthermore, postmenopausal women were women who had no menstrual period in the past 12 months, except for cessation of menstruation due to pregnancy, breastfeeding, and other medical conditions/treatments [23]. Female hormones in the form of pills, patches, cream, suppository, or injection were used. The types of hormone pills were estrogen only (e.g., Premarin), progestin only (e.g., Provera), or estrogen/progestin combo pills (e.g., Prempro/Premphase), and the types of hormone patches were estrogen only or estrogen/progestin combo patches. Data on only “yes” responses to female hormone use, vigorous activities, moderate activities, arthritis, thyroid disease, liver disease, kidney disease, and diabetes are included with other participant characteristics in Table 1.

**Table 1:** Weighted characteristics of the study population based on age at menarche

Characteristics	Age at menarche (years)			*P* value
	≤ 12 (*n* = 570)	13–15 (*n* = 538)	≥ 16 (*n* = 87)	
Age (years), mean (95% CI)	53.02 (52.45, 53.58)	53.77 (53.26, 54.27)	52.30 (51.06, 53.55)	0.095
Years since menopause (years), mean (95% CI)	8.68 (7.88, 9.48)	7.77 (6.99, 8.55)	9.03 (7.38, 10.68)	0.088
Body mass index (kg/m^2^), mean (95% CI)	30.83 (29.97, 31.68)	29.23 (28.21, 30.24)	27.89 (26.36, 29.41)	< 0.001
Lumbar spine BMD (g/cm^2^), mean (95% CI)	1.00 (0.98, 1.02)	0.98 (0.96, 1.00)	0.92 (0.89, 0.95)	< 0.001
Race, percent (95% CI)				0.136
Non-Hispanic white	67.53 (61.38, 73.14)	64.66 (58.13, 70.70)	57.01 (42.03, 70.81)	
Non-Hispanic black	12.64 (9.77, 16.21)	13.82 (10.54, 17.91)	18.44 (11.48, 28.28)	
Mexican American	7.39 (5.28, 10.25)	6.22 (4.35, 8.81)	3.23 (1.24, 8.16)	
Other race	12.43 (9.46, 16.18)	15.30 (12.36, 18.78)	21.32 (12.52, 33.90)	
Education level, percent (95% CI)				0.586
Less than high school	12.05 (8.72, 16.41)	13.30 (9.64, 18.07)	14.10 (6.72, 27.20)	
High school	26.72 (22.36, 31.58)	22.31 (16.98, 28.74)	30.89 (17.77, 48.03)	
More than high school	61.24 (54.70, 67.39)	64.39 (56.84, 71.29)	55.01 (38.36, 70.61)	
Family PIR, percent (95% CI)				0.269
< 1.3	16.59 (13.21, 20.63)	18.28 (13.72, 23.94)	16.30 (8.63, 28.65)	
1.3–3.5	31.51 (26.93, 36.48)	25.27 (21.23, 29.78)	41.44 (25.71, 59.13)	
≥ 3.5	45.05 (38.87, 51.39)	49.76 (42.18, 57.36)	38.34 (23.71, 55.43)	
Missing	6.85 (4.56, 10.17)	6.69 (4.36, 10.13)	3.93 (1.57, 9.52)	
Total calcium (mmol/L), percent (95% CI)				0.003
*T*_1_ (1.900–2.300)	33.63 (28.74, 38.88)	23.74 (19.22, 28.94)	30.13 (18.63, 44.83)	
*T*_2_ (2.301–2.375)	33.94 (28.93, 39.34)	38.41 (33.76, 43.28)	19.12 (11.32, 30.44)	
*T*_3_ (2.376–2.750)	30.05 (24.95, 35.70)	34.39 (28.92, 40.32)	41.73 (26.97, 58.15)	
Missing	2.38 (1.15, 4.87)	3.45 (1.61, 7.24)	9.01 (3.43, 21.65)	
Serum phosphorus (mmol/L), percent (95% CI)				0.070
*T*_1_ (0.743–1.130)	24.48 (20.86, 28.50)	32.16 (26.08, 38.91)	22.51 (12.88, 36.34)	
*T*_2_ (1.131–1.259)	34.16 (28.19, 40.68)	26.62 (21.02, 33.10)	37.36 (21.93, 55.88)	
*T*_3_ (1.260–1.905)	39.05 (33.43, 44.98)	37.76 (31.72, 44.21)	31.11 (17.54, 48.95)	
Missing	2.31 (1.10, 4.79)	3.45 (1.61, 7.24)	9.01 (3.43, 21.65)	
Smoking behavior, percent (95% CI)				0.749
Never	56.92 (51.34, 62.34)	57.68 (50.46, 64.59)	60.74 (43.71, 75.50)	
Former	19.64 (15.39, 24.72)	21.58 (16.69, 27.43)	24.43 (12.36, 42.56)	
Current	23.44 (18.74, 28.90)	20.74 (16.35, 25.95)	14.83 (5.68, 33.48)	
Age started smoking, mean (95% CI)	18.21 (17.17, 19.25)	18.17 (17.26, 19.08)	19.20 (16.13, 22.26)	0.776
Alcohol consumption, percent (95% CI)				0.584
Non-drinker	14.68 (11.26, 18.92)	14.91 (11.37, 19.30)	27.02 (15.04, 43.64)	
Moderate drinker	34.51 (29.39, 40.02)	34.27 (27.28, 42.02)	23.86 (12.31, 41.14)	
Heavy drinker	39.54 (33.96, 45.40)	37.90 (31.52, 44.71)	36.88 (22.48, 54.07)	
Missing	11.27 (8.33, 15.08)	12.93 (9.20, 17.86)	12.24 (4.01, 31.76)	
Parity, percent (95% CI)				0.026
0–1	16.78 (12.61, 21.99)	19.08 (15.12, 23.78)	22.38 (10.67, 41.04)	
2–3	62.66 (58.13, 66.97)	49.65 (44.39, 54.92)	52.05 (36.58, 67.13)	
≥ 4	9.79 (7.56, 12.60)	14.52 (10.96, 18.99)	8.30 (3.49, 18.45)	
Missing	10.77 (7.27, 15.67)	16.75 (12.01, 22.88)	17.28 (7.53, 34.90)	
Female hormone use, percent (95% CI)	28.67 (23.66, 34.26)	26.01 (20.79, 32.00)	25.60 (14.02, 42.05)	0.750
Vigorous activities, percent (95% CI)	29.65 (24.72, 35.10)	25.68 (20.64, 31.46)	32.53 (17.51, 52.28)	0.525
Moderate activities, percent (95% CI)	60.75 (53.61, 67.47)	67.11 (61.76, 72.06)	64.24 (47.20, 78.32)	0.290
Arthritis, percent (95% CI)	40.67 (35.38, 46.19)	36.13 (29.39, 43.46)	27.43 (15.60, 43.61)	0.177
Thyroid disease, percent (95% CI)	22.76 (18.59, 27.56)	16.11 (12.08, 21.16)	25.12 (12.19, 44.76)	0.129
Liver disease, percent (95% CI)	3.60 (2.35, 5.47)	5.93 (3.13, 10.95)	9.66 (2.81, 28.34)	0.218
Kidney disease, percent (95% CI)	2.55 (1.25, 5.15)	2.83 (1.21, 6.49)	1.26 (0.26, 5.81)	0.782
Diabetes, percent (95% CI)	11.81 (8.37, 16.42)	10.59 (7.30, 15.12)	4.72 (1.94, 11.04)	0.496

*CI* confidence interval, *BMD* bone mineral density, *PIR* poverty income ratio, *T* tertile

### Statistical methods

Due to the complex sampling design of the NHANES, appropriate sample weights were considered in all analyses. All analyses were performed using package R (https://www.r-project.org/) and EmpowerStats software (http://www.empowerstats.net/cn/). Statistical significance was set for all tests as a *P* value < 0.05. The results for continuous variables are reported as mean (95% confidence interval [CI]) and that for categorical variables are reported as percent (95% CI). For continuous and categorical variables, *P* values were computed using the weighted linear regression model and chi-square test, respectively. A weighted multivariate linear regression model was used to evaluate the association between AAM and LS BMD. Due to the great effect of female hormones on BMD, we conducted a sensitivity analysis on participants who did not use female hormones. In addition, a generalized additive model and smooth curve fitting were used to explore the relationship between AAM and LS BMD.

## Results

A total of 1195 postmenopausal women aged 40–59 were included in the analysis (Fig. 1). The weighted characteristics of the study population based on AAM are presented in Table 1. BMI, total calcium, parity, and LS BMD were significantly different among the three AAM categories (menarche age: ≤ 12, 13–15, and ≥ 16 years).

**Fig. 2:**
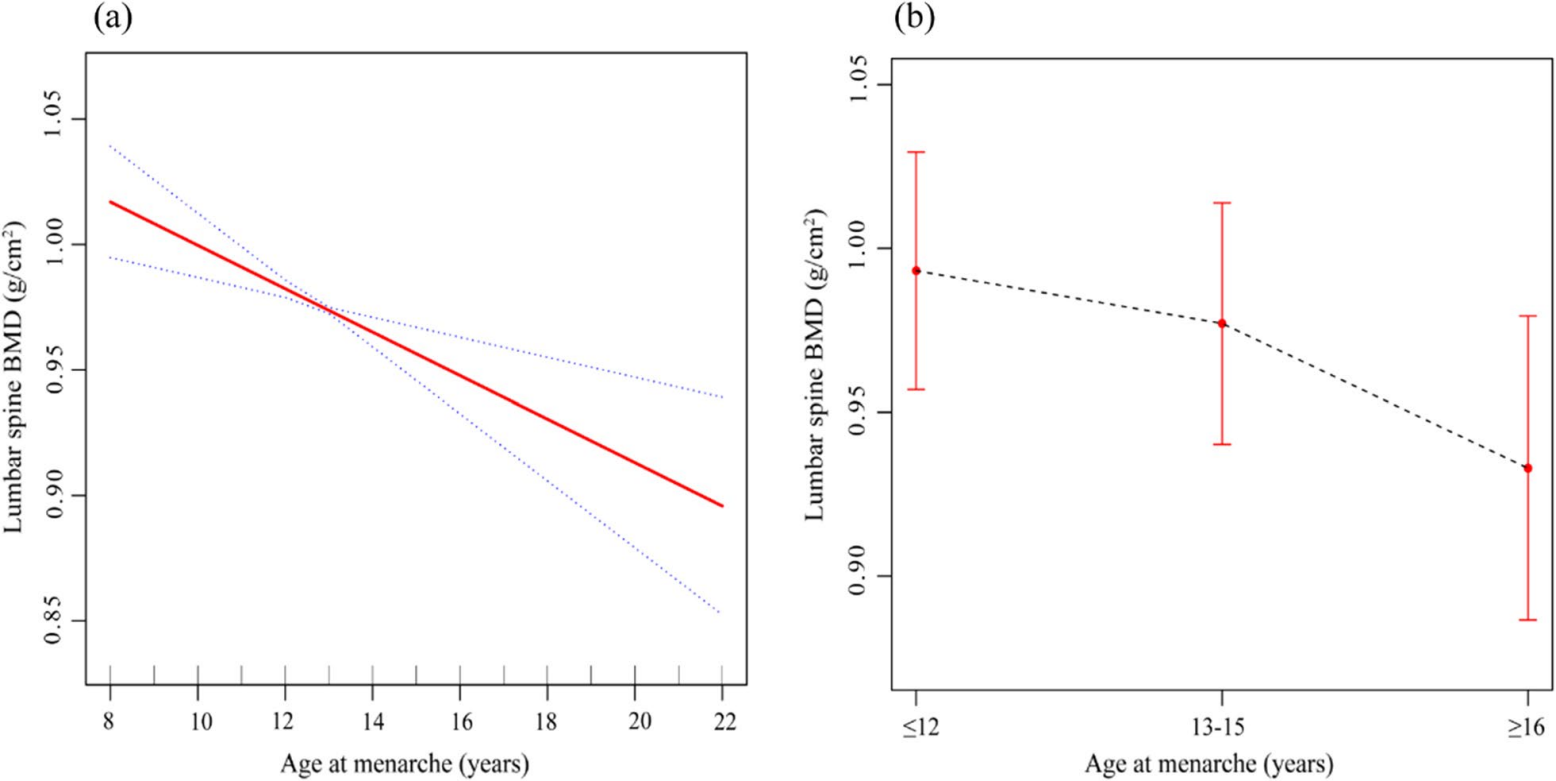
Association between age at menarche and lumbar spine BMD. **a** Menarche age as a continuous variable. The solid red line represents the fitting line between variables, and the dotted blue lines represent the 95% confidence interval. **b** Menarche age as a categorical variable. The figure shows means and 95% confidence intervals. Age, race, BMI, education level, family PIR, smoking behavior, alcohol consumption, years since menopause, parity, female hormone use, vigorous activities, moderate activities, total calcium, serum phosphorus, arthritis, thyroid disease, liver disease, kidney disease, and diabetes were adjusted for.

Table 2 shows the linear regression analyses results. In the unadjusted model, a menarche age of ≥ 16 years compared with a menarche age of ≤ 12 years was associated with lower LS BMD (*β* = − 0.083, 95% CI − 0.117, − 0.048, *P* < 0.001). After adjusting for potential confounding factors, there was still a negative correlation in model 2 (*β* = − 0.078, 95% CI − 0.113, − 0.042, *P* < 0.001) and model 3 (*β* = − 0.065, 95% CI − 0.096, − 0.033, *P* < 0.001). This significant relationship persisted after excluding participants who used female hormones (*β* = − 0.053, 95% CI − 0.089, − 0.016, *P* = 0.006) (Table 3). Moreover, the negative correlations between AAM and LS BMD are more intuitively presented in Fig. 2.

**Table 2:** Associations between age at menarche and lumbar spine BMD

Age at menarche (years)	Beta	SE	*P* value	95% CI
Model 1				
≤ 12	Reference			
13–15	− 0.020	0.014	0.160	(− 0.046, 0.007)
≥ 16	− 0.083	0.018	< 0.001	(− 0.117, − 0.048)
Model 2				
≤ 12	Reference			
13–15	− 0.013	0.014	0.355	(− 0.041, 0.015)
≥ 16	− 0.078	0.018	< 0.001	(− 0.113, − 0.042)
Model 3				
≤ 12	Reference			
13–15	− 0.014	0.014	0.322	(− 0.041, 0.013)
≥ 16	− 0.065	0.016	< 0.001	(− 0.096, − 0.033)

Model 1: no covariates were adjusted for

Model 2: age, race, and BMI were adjusted for

Model 3: age, race, BMI, education level, family PIR, smoking behavior, alcohol consumption, years since menopause, parity, female hormone use, vigorous activities, moderate activities, total calcium, serum phosphorus, arthritis, thyroid disease, liver disease, kidney disease, and diabetes were adjusted for

**Table 3:** Associations between age at menarche and lumbar spine BMD excluding individuals who used female hormones (*n* = 248)

Age at menarche (years)	Beta	SE	*P* value	95% CI
≤ 12	Reference			
13–15	− 0.012	0.016	0.460	(− 0.044, 0.020)
≥ 16	− 0.053	0.018	0.006	(− 0.089, − 0.016)

Age, race, BMI, education level, family PIR, smoking behavior, alcohol consumption, years since menopause, parity, vigorous activities, moderate activities, total calcium, serum phosphorus, arthritis, thyroid disease, liver disease, kidney disease, and diabetes were adjusted for

## Discussion

The present study examined the association of AAM with LS BMD in postmenopausal women. The main finding of the study was that an older menarche age was associated with lower BMD values at the LS region in postmenopausal women after adjusting for age, race, BMI, education level, family PIR, smoking behavior, alcohol consumption, years since menopause, parity, female hormone use, vigorous activities, moderate activities, total calcium, serum phosphorus, arthritis, thyroid disease, liver disease, kidney disease, and diabetes.

BMD is a non-invasive measure of bone health and is commonly used in clinical practice to diagnose osteoporosis and osteopenia. According to the diagnostic criteria recommended by the World Health Organization [24], low bone density was defined as: (1) osteopenia: a BMD value between 1.0 and 2.5 standard deviations below the mean of the young adult reference group and (2) osteoporosis: a BMD value of 2.5 or more standard deviations below the mean of the young adult reference group. Among postmenopausal women, osteoporosis was one of the most common bone diseases, leading to bone fragility and increased risk of fracture. The spine was the most common first fracture event in postmenopausal women [25], and vertebral fractures were associated with increased mortality [26], reduced lung volume and height [27], and decreased quality of life [28]. A meta-analysis showed a 1.3-fold increased risk of vertebral fracture in women for a decrease in LS BMD of one standard deviation below the age-adjusted mean [29]. Therefore, it is particularly important to identify the risk factors leading to low BMD and thus focus on bone health care.

Previous studies have reported conflicting findings. Some studies, which are similar to ours, noted that late AAM was associated with decreased BMD and a risk of subsequent osteoporosis or osteoporotic fractures. Zhang et al. found in a Mendelian randomization study that females with later menarche age were at greater risk of low LS BMD and suggested appropriate preventive and protective strategies to reduce spinal bone loss in menopausal women [30]. Another study from China showed that AAM was negatively correlated with LS BMD in elderly women [31]. A cohort study in Japan found an independent and significant association between late menarche and the risk of vertebral fracture in later life [32]. Furthermore, a British birth cohort study showed that later menarche age was correlated with lower LS aBMD and confirmed that the association between later puberty and lower BMD persists into early old age [33]. In contrast, other studies have found no correlation between AAM and LS BMD in postmenopausal women. Gerdhem et al. reported no effect of AAM on LS BMD in older women, and after further quartiles of AAM, no statistical difference was found in the largest quartile compared to the others [17]. In addition, Hagemans et al. [15] and Hassa et al. [16] concluded that AAM had no effect on postmenopausal LS BMD. Years since menopause, inclusion criteria, selection of confounders, and representation of the population may explain the differences between the studies, but more research on the underlying mechanisms is still needed to explain this phenomenon.

Studies have shown that AAM is related to bone density and bone metabolism in a certain connection [34]. This may be because women with a later menarche age have a shorter duration of exposure to estrogen, which is a vital hormonal factor in the formation and growth of women’s bones [35]. A recent article also confirmed the close connection between estrogen 17 β-estradiol and bone density [36]. Another study showed that females with late menarche may achieve lower peak bone mass at certain skeletal sites, which may increase their susceptibility to osteoporosis in later life [37]. Therefore, the earlier AAM, the earlier the level of estrogen gets close to that of adults and the earlier it plays a role in promoting the formation of bone by osteoblasts and inhibiting the absorption of bone by osteoclasts. This ensures that women obtain higher peak bone mass and will reduce the risk of osteoporosis in middle-aged and elderly. The mechanisms of the effect of menarche age on LS BMD are complex, and further basic research is needed in the future to confirm this relationship.

In this study, we used appropriate weights to analyze a representative sample of a multi-ethnic population according to the analytical guidelines of the NHANES. Furthermore, detailed demographics, examination, laboratory, and questionnaire data allowed us to adjust for more comprehensive confounders in the multivariate regression analysis to obtain a more objective statistical relationship between AAM and LS BMD. However, there were several limitations in this study. First, this study did not evaluate BMD at other skeletal sites associated with postmenopausal osteoporosis, such as the femoral neck and total hip, because only LS BMD data were complete in the four cycles of the NHANES 2011–2018. Second, there may be other potential confounding factors that were not considered in this study. Third, some of the collected data were self-reported, which may lead to recall bias. Further prospective studies with larger samples and more skeletal sites are necessary.

Overall, our study found that postmenopausal women with a menarche age of ≥ 16 years had significantly lower LS BMD than that had by those with a menarche age of ≤ 12 years. As a result of this study, postmenopausal women with a late menarche age may have a higher risk of lumbar osteoporotic fractures and need better bone health care.

## Data Availability

The data of this paper were obtained from the public database (https://www.cdc.gov/nchs/nhanes/).

## Abbreviations

BMD: Bone mineral density
AAM: Age at menarche
LS: Lumbar spine
NHANES: National Health and Nutrition Examination Survey
BMI: Body mass index
DXA: Dual-energy X-ray absorptiometry
PIR: Poverty income ratio
CI: Confidence interval
